# Advancements and Impacts of Assistive Technologies, Robotics, and Automated Machines in Healthcare: Insights from an Editorial Initiative of Exploration

**DOI:** 10.3390/healthcare13101121

**Published:** 2025-05-12

**Authors:** Daniele Giansanti

**Affiliations:** Centre Tisp, Istituto Superiore di Sanità, 00100 Rome, Italy; daniele.giansanti@iss.it; Tel.: +39-06-4990-2701

Assistive technologies, robotics, and automated machines are revolutionizing the healthcare sector, offering groundbreaking solutions that enhance patient outcomes and improve operational efficiency [1]. These technologies empower individuals with disabilities to live more independently and assist healthcare professionals in performing complex tasks. For instance, AI-integrated assistive devices, such as smart wheelchairs and exoskeletons, enhance mobility and autonomy for users [2]. Robotic applications in healthcare encompass surgical procedures, rehabilitation, remote patient monitoring, and social assistance, reshaping the delivery of healthcare across various domains [3]. The integration of automated systems into clinical workflows enables more accurate decision-making, reduces human error, and streamlines processes, thereby improving efficiency and reliability [4]. Assistive technologies also promote greater inclusion by enabling individuals to participate fully in daily activities, utilizing tools ranging from communication aids to mobility devices [5]. The COVID-19 pandemic has further highlighted the critical role of robotics in healthcare, with robots facilitating tasks such as disinfection, the delivery of medicines, and telemedicine services [6]. As healthcare systems evolve, the strategic implementation of these technologies is crucial for advancing personalized care, improving patient experiences, and ensuring sustainability in healthcare practices [7]. Their potential to transform both individual and systemic healthcare experiences underscores their importance as we look toward the future of medicine [8].

In order to explore the evolving landscape of research in this field, and following the success of the first edition [9], we decided to launch a second Special Issue intended [10] to serve as a scientific forum for international scholars to meet and exchange ideas.

This Special Issue, in addition to this concluding editorial, includes 14 contributions (Contribution 1–14) which comprise an introductory editorial (Contribution 1), eight research articles (Contribution 2–9), three reviews (Contribution 10–12), a systematic review (Contribution 13), and a study protocol (Contribution 14).

- *Outcome from the Scientific Articles*

Table 1 provides a concise overview of the research articles featured in this Special Issue, all of which focus on various aspects of digital health and technological innovations in healthcare. These studies explore the intersection of advanced technologies, user engagement, and clinical practices, reflecting the enhancement of personalized care, digital literacy, and the integration of AI and robotics into health systems.

The articles included in this Special Issue cover a range of topics, including the use of wearable exoskeletons that reduce physical fatigue and improve gait dynamics (Contribution 2), the factors that influence the engagement of users with mobile health applications through the Technology Acceptance Model (Contribution 3), and the importance of e-health literacy during the COVID-19 pandemic and its improvement of public health resilience (Contribution 4). Other notable contributions examine how AI, such as ChatGPT, can enhance medical communication for patients, highlighting both its potential and limitations (Contribution 5), and explore personalized neurorehabilitation strategies for spinal cord injury patients, emphasizing tailored treatment pathways (Contribution 6).

Additionally, this Special Issue includes studies on the acceptance of social robots by older adults (Contribution 7), the challenges associated with engaging adolescents in health-promoting behaviors through mobile apps (Contribution 8), and insights from Israeli nurses on the effectiveness of telenursing during the pandemic (Contribution 9). Together, these articles provide an in-depth exploration of this field of, with a focus on improving patient outcomes, enhancing accessibility, and fostering engagement with digital health tools.

- *Outcome from the Other Contributions*

Table 2 presents a summary of other articles published in the collection. The first three are reviews (Contributions 10–12). The first study (Contribution 10) is a review that examines the use of Transoral Robotic Surgery (TORS) as a de-escalation strategy in the treatment of OPSCC, highlighting the benefits of reducing additional treatments without compromising oncological outcomes. The second review (Contribution 11) explores nursing competencies in robotics, identifying 17 key areas in which competence is required for adapting robotics in nursing. Finally, the third review (Contribution 12) analyzes recent advancements in teledermatology (TD) and mHealth, emphasizing how the COVID-19 pandemic accelerated the adoption of remote care solutions, while also addressing challenges related to data privacy and regulation. These reviews underscore the importance of technology in improving the efficiency and accessibility of healthcare, while highlighting issues related to training and regulatory management. This Special Issue also includes a systematic review (Contribution 13) that identifies significant gaps in the literature regarding robotic rehabilitation, particularly the lack of standardized tools for assessing patient needs using the ICF framework, and highlighting the importance of developing tailored surveys that better evaluate patients with sensory, motor, and cognitive disorders. It concludes with a study protocol (Contribution 14) that present the FIT4TeleNEURO trial, which investigates the effectiveness of early rehabilitation interventions via mixed-model telerehabilitation protocols for patients with Parkinson’s disease and multiple sclerosis; this aims to provide sustainable solutions for early and continuous rehabilitation in real-life care settings.

- *Conclusions and Future Routes*

In conclusion, the studies presented in this Special Issue emphasize the transformative potential of assistive technologies, automated machines, and robotics in healthcare (Contributions 2–14). Wearable exoskeletons (Contribution 2) are able to reduce physical fatigue and improve mobility, while robotic rehabilitation systems (Contribution 13) highlight the need for standardized tools that better assess patient needs using frameworks such as the ICF. These gaps suggest that there is a need for more tailored, patient-centered assessments in robotic rehabilitation. The integration of advanced data analysis in neurorehabilitation (Contribution 6) is also crucial, highlighting the shift toward personalized care. Additionally, the FIT4TeleNEURO trial (Contribution 14) explores the role of telerehabilitation in early intervention for chronic diseases such as Parkinson’s and multiple sclerosis, emphasizing the potential for scalable, remote solutions. 

Future research should focus on refining these technologies, enhancing user acceptance, and addressing the gaps observed in nursing competencies for robotics (Contribution 11). Moreover, expanding the use of telehealth systems, as seen in teledermatology (Contribution 12), will enhance accessibility and reduce healthcare disparities. Ultimately, the continued development and integration of robotics and automation in healthcare could offer more efficient, personalized care and improved outcomes for a diverse range of patients.

## Figures and Tables

**Table 1:** Outline of scientific articles.

References	Brief Summary	Focus	Contribution to Healthcare
(Contribution 2)	This study evaluates the biomechanical impact of wearing a lower-limb exoskeleton in healthy individuals, using EMG and gait analysis tools to assess changes in muscle activity, fatigue levels, and gait parameters. The results show reduced muscle strain and improved gait dynamics, offering a foundation for the broader application of wearable robotics.	Explores the integration of wearable robotic systems in rehabilitation and occupational health, focusing on how assistive exoskeletons can support motor function, reduce strain, and enhance mobility in both therapeutic and industrial settings.	Demonstrates how exoskeletons can reduce physical fatigue and optimize movement patterns, reinforcing their value in both clinical rehabilitation programs and preventive strategies in physically demanding occupations.
(Contribution 3)	This study applies the Technology Acceptance Model (TAM) to analyze user interactions with mobile health (mHealth) applications. It explores how these tools’ perceived ease of use, usefulness, and user satisfaction influence the intention to continue using them, with implications for long-term digital health engagement.	Investigates the behavioral and psychological factors influencing user interaction with mHealth solutions, with a specific focus on usability, perceived benefits, and motivational elements that drive sustained engagement with digital health platforms.	Enhances our understanding of the psychological and usability factors driving mHealth adoption, offering actionable insights for designing apps that are more likely to be embraced and sustained by diverse user populations.
(Contribution 4)	This study investigates the levels of e-health literacy among Chinese university students during the COVID-19 pandemic, identifying factors such as academic discipline, gender, and online behavior that shape effective health information-seeking practices.	Focuses on the role of digital health literacy in managing public health crises, examining how individuals access, interpret, and use online health information, and identifying educational gaps and demographic patterns in digital competency.	Highlights the need for targeted e-health literacy programs to support critical appraisal and the responsible use of online health content, thereby contributing to public health resilience during emergencies.
(Contribution 5)	This study analyzes ChatGPT’s ability to explain glomerular disorder treatments to lay audiences. It compares the readability and accuracy of responses at general versus simplified levels, finding potential for improving medical communication through AI with caution regarding accuracy.	Examines the potential of generative AI tools for health communication, especially their capacity to deliver complex medical content in an accessible format, bridging the gap between professional knowledge and patient understanding.	Suggests that conversational AI tools such as ChatGPT could democratize access to complex medical information, provided there is oversight to ensure clarity without compromising medical precision.
(Contribution 6)	This study introduces a clustering-based approach to compare traditional and technology-assisted neurorehabilitation in patients with spinal cord injury. It emphasizes how functional and neurological profiles can inform tailored therapy selection for better outcomes.	Focuses on the integration of advanced data analysis and profiling techniques in neurorehabilitation to customize treatment paths, emphasizing a shift from standardized to personalized therapeutic approaches.	Supports precision rehabilitation by showing how data-driven classification can match patients to the most suitable therapeutic approaches, enhancing the efficacy of tech-integrated interventions.
(Contribution 7)	This is a conceptual study combining behavioral theories to explore psychological and contextual factors influencing older adults’ acceptance of social robots. It addresses emotional, functional, and cognitive aspects of human-robot interaction.	Investigates how theoretical models of technology acceptance apply to the use of social and assistive robotics among the elderly, emphasizing cognitive engagement, emotional response, and perceived usefulness in daily life support.	Provides a multidimensional framework for designing socially acceptable robots that promote autonomy, emotional comfort, and social connection among the elderly.
(Contribution 8)	This study assesses adolescents’ interaction with the “Healthy Jeart” mobile app designed to promote healthy lifestyles. The study identifies usability barriers and engagement issues that affect sustained use and behavior change.	Examines the specific usability needs and behavioral preferences of adolescents in mHealth app design, focusing on how digital tools can better capture and retain teen engagement in preventive health practices.	Offers valuable design recommendations for creating youth-friendly health apps, which can play a key role in early prevention and health promotion among teenagers.
(Contribution 9)	This study is a survey-based study on Israeli nurses’ experiences with telenursing during COVID-19, comparing perceptions of quality, communication, and trust in remote versus in-person care.	Explores frontline healthcare providers’ perspectives on the delivery of remote care, focusing on how communication quality, professional confidence, and patient interaction are shaped by telehealth environments.	Underscores the importance of training, communication protocols, and digital empathy to ensure that telehealth maintains clinical effectiveness and supports patient–nurse relationships.

**Table 2:** Outline of the other contributions.

References/ Study Category	Brief Summary	Focus	Contribution to Healthcare
(Contribution 10)/ Review	This paper evaluates the role of Transoral Robotic Surgery (TORS) as a de-escalation strategy for managing HPV-related oropharyngeal squamous cell carcinoma (OPSCC). The review covers clinical trials and the outcomes associated with TORS, focusing on reducing treatment-related side effects while maintaining oncological control.	Focuses on the use of TORS in the management of HPV-positive OPSCC as a means of reducing morbidity while preserving oncological outcomes. It emphasizes the importance of risk stratification and HPV status in treatment decisions.	TORS presents a viable treatment strategy for HPV-related OPSCC by reducing the need for adjuvant therapy, minimizing side effects, and maintaining high tumor control and survival rates. This method offers a new direction for de-escalation in cancer treatment, enhancing the quality of life for patients.
(Contribution 11)/ Review	This paper reviews the development of nursing robotic competencies between 2017 and 2023, identifying 17 competencies across five categories: assessment, diagnosis, planning, intervention, and evaluation. It highlights the gap in nursing robotic competencies and the need for further advances in nursing informatics and robotics to meet healthcare demands.	Examines the integration of robotics into nursing and the competencies needed for effective use, focusing on technological advancements and their impact on nursing practice.	This study identifies the key competencies required for the use of robotics in nursing, advocating for further training and role definition in the field of nursing informatics. It suggests a need to adapt nursing education to integrate new technologies for improving care and meeting evolving healthcare needs.
(Contribution 12)/ Review	This narrative review explores the advancements in mobile health (mHealth) in tele-dermatology (TD) post-COVID-19, focusing on the integration of AI, wearable sensors, and mobile apps. It discusses the benefits of this integration, such as improved service quality, reduced healthcare costs, and increased accessibility, while also addressing challenges such as ethics and data privacy.	Focuses on rapid advancements in mHealth in tele-dermatology during the COVID-19 pandemic, particularly the integration of AI and mobile technologies in dermatology.	mHealth in tele-dermatology has the potential to revolutionize healthcare delivery by enabling remote monitoring, improving accessibility, and empowering patients. This development helps reduce the burden on healthcare systems while maintaining high-quality care, particularly in underserved areas.
(Contribution 13)/ Systematic Review	This systematic review explores gaps in current robotic rehabilitation literature, particularly regarding tools for assessing patients’ needs based on the ICF framework. It analyzes 39 studies and finds a reliance on semi-structured interviews without standardized tools, highlighting a need for tailored surveys to evaluate sensory, motor, and cognitive needs.	Focuses on the integration of robotic rehabilitation into clinical practice and emphasizes the importance of standardized ICF-based assessment tools. It identifies gaps in evaluating conditions such as Parkinson’s disease and frailty.	This study contributes by advocating for the development of standardized, ICF-based tools to improve patient-centered robotic rehabilitation strategies, enhancing the effectiveness of rehabilitation for patients with various health conditions.
(Contribution 14)/ Study protocol	The FIT4TeleNEURO trial investigates the effectiveness of telerehabilitation (TR) protocols in early rehabilitation for Parkinson’s disease and multiple sclerosis patients, comparing it with conventional treatments. It involves 300 patients and evaluates static and dynamic balance, motor function, and quality of life.	Focuses on evaluating the effectiveness of telerehabilitation in chronic neurological diseases, specifically comparing different TR protocols to conventional care. It looks at how TR can improve early rehabilitation availability and outcomes.	This trial contributes to healthcare by exploring scalable telerehabilitation solutions that could offer more targeted, efficient, and accessible rehabilitation, particularly for early intervention in chronic neurological diseases such as Parkinson’s and multiple sclerosis.
